# Feasibility of a machine learning model for predicting weight gain after kidney transplantation machine learning and weight gain after kidney transplantation

**DOI:** 10.1371/journal.pone.0360501

**Published:** 2026-10-22

**Authors:** Júlia de Melo Cardoso de Freitas, Pedro Lemos Ballester, Elis Forcellini Pedrollo, Camila Corrêa, Rafaella Câmara Rocha Menezes, Cristiane Bauermann Leitão, Gabriela Corrêa Souza

**Affiliations:** 1 Post-Graduate Program in Medical Sciences: Endocrinology, Universidade Federal do Rio Grande do Sul, Porto Alegre, Rio Grande do Su, Brazil; 2 Post-Graduate Program in Neuroscience, McMaster University, Hamilton, Ontario, Canada; 3 Nutrition and Dietetics Division, Hospital de Clínicas de Porto Alegre, Porto Alegre, Rio Grande do Su, Brazil; 4 Post-Graduate Program in Health Sciences, Universidade Federal de Ciências da Saúde de Porto Alegre, Porto Alegre, Rio Grande do Su, Brazil; 5 Department of Internal Medicine, School of Medicine, Universidade Federal do Rio Grande do Sul, Porto Alegre, Brazil; 6 Endocrine Division, Hospital de Clínicas de Porto Alegre, Porto Alegre, Brazil; 7 Department of Nutrition, School of Medicine, Universidade Federal do Rio Grande do Sul, Porto Alegre, Brazil; 8 Post-Graduate Program in Food, Nutrition and Health, School of Medicine, Universidade Federal do Rio Grande do Sul, Porto Alegre, Brazil; Phramongkutklao College of Medicine, THAILAND

## Abstract

**Background:**

Excessive weight gain after kidney transplantation adversely affects patient and graft outcomes. Machine Learning has been increasingly used to improve risk prediction across a range of transplant-related outcomes, yet its application to post-transplant weight trajectories remains unexplored. This study is the first to evaluate the feasibility of using Machine Learning algorithms to identify predictors of weight gain after kidney transplantation.

**Methods:**

This retrospective cohort study analyzed 374 adult kidney transplant recipients. We developed an Elastic Net regression model with 10-fold cross-validation to predict body weight variation at 12 months post-transplant. The analysis screened a comprehensive set of demographic and clinical variables, including recipient characteristics, donor type, dialysis duration, and pre-transplant hypertension, to identify predictors within the training (75%) and testing (25%) datasets.

**Results:**

Mean weight gain was 4.0 ± 7.4 kg. The Elastic Net model retained four predictors in the final model: female sex, deceased donor source, age, and pre-transplant body mass index. However, the model demonstrated limited predictive performance (R^2^ = 0.10; Mean Absolute Error = 8.7%), indicating that baseline variables explain only a small fraction of the observed variability in weight change.

**Conclusions:**

This pioneering study establishes an analytical benchmark and reveals the informational limits of standard clinical datasets for predicting post-transplant weight gain. While Machine Learning application is feasible, current demographic and clinical variables are insufficient for precise individual prediction. Future models must integrate more granular behavioral and pharmacological data to achieve clinically actionable risk stratification.

## Introduction

Kidney transplantation is the preferred therapeutic option for patients with end-stage renal disease, offering superior cost-effectiveness, enhanced survival, and improved quality of life compared with dialysis modalities [1]. Despite these well-established benefits, comorbidities are highly prevalent in renal graft recipients, and their implications for graft function and long-term outcomes remain incompletely understood [2]. Metabolic risk factors for cardiovascular disease, such as impaired glucose homeostasis, dyslipidemia, metabolic syndrome, and obesity are highly prevalent in the post-transplant setting and may adversely affect postoperative recovery and compromise graft survival [3].

Excessive weight gain is frequently observed following kidney transplantation and has been associated with an increased risk of systemic arterial hypertension, hyperlipidemia, post-transplant diabetes mellitus, impaired graft function, and cardiovascular disease [4–7]. In a retrospective study conducted in Brazil by our research group involving 374 patients, 31.2% and 20.9% exhibited an increase of at least 10% and 5% of their baseline body weight, respectively, within the first year post-transplantation [8]. Although that study identified the prevalence of post-transplant weight gain and associated risk factors using conventional statistical analyses, it was not designed to develop or evaluate predictive models. An average weight gain of approximately 10% relative to pre-transplant values during the first year after transplantation has been consistently reported [9]. This increase may be influenced by immunosuppressive therapy, particularly due to drug-induced hyperphagia, as well as behavioral factors, such as increased caloric intake and reduced physical activity [10]. In addition, non-modifiable determinants, including genetic predisposition and individual characteristics, also may play a role [11,12]. Furthermore, other factors have been identified as contributors to post-transplant weight gain, including self-reported race/ethnicity (Black), younger age at transplantation, female sex, dialysis modality, and tacrolimus use [12–15].

Given this multifactorial nature, identifying and quantifying the contribution of these variables to post-transplant weight gain represents a significant challenge. Traditional statistical approaches may be limited in their ability to capture complex clinical phenomena, as they generally rely on predefined functional forms, assume linear relationships, and require explicit specification of interaction terms [16,17]. It should be acknowledged that more advanced statistical approaches, such as generalized additive models and penalized regression techniques, can partially accommodate nonlinear effects and interactions. However, these methods generally require prior specification of model structure and may remain limited when underlying relationships are highly complex or not known a priori [17].

In multifactorial biological systems, where nonlinear and interdependent mechanisms are common, these constraints may hinder the identification of relevant patterns. Machine learning (ML), a set of data-driven computational methods that enable pattern recognition and predictive modeling without explicit specification of underlying relationships, provides greater flexibility by modeling nonlinear relationships and capturing interactions among predictors [16–18]. In this context, ML-based prediction models may offer a flexible framework for risk stratification and support the identification of patients at higher risk for excessive weight gain following transplantation [16].

Considering the capacity of ML algorithms to handle large datasets and to construct predictive models for complex outcomes, such as post-transplant weight gain, these tools may support health professionals in developing care protocols aimed at preventing adverse outcomes in clinical practice [16]. Among these methods, the Elastic Net algorithm has been shown to be effective in identifying direct and independent associations between outcomes and predictor variables, while simultaneously accounting for multicollinearity and adjusting for relevant confounders [18]. It is particularly suitable for clinical datasets with correlated predictors, as it allows for simultaneous variable selection and coefficient shrinkage. Previous studies have primarily focused on identifying factors associated with post-transplant weight gain using conventional statistical approaches. However, whether ML-based predictive models are feasible for this outcome remains unknown. Therefore, the objective of this study was to evaluate the feasibility of developing an ML-based model to predict percentage body weight variation at 12 months after kidney transplantation and to identify the predictors retained in the final model.

## Methods

### Study design and population

This retrospective cohort study was conducted at the Hospital de Clínicas de Porto Alegre, Brazil, and included kidney transplant recipients between January 2006 and July 2013. Patients aged 18 years or older were eligible. Exclusion criteria were previous diagnosis of diabetes mellitus, graft loss or death within the first post-transplant year, retransplantation or multiple organ transplantation, and absence of weight data at follow-up points.

Clinical and demographic variables were extracted from medical records, including age at transplantation, sex, race/skin color, etiology of chronic kidney disease, comorbidities (including hypertension, defined as a documented medical diagnosis before transplantation), family history of diabetes mellitus, dialysis modality and duration, and donor characteristics, including donor sex and donor type (living or deceased). Anthropometric assessments included pre-transplant dry weight and body weight (kg) at 3, 6, and 12 months post-transplantation, with body mass index (BMI) calculated according to World Health Organization cut-off points. Weight gain was evaluated both as absolute change in kilograms and as percentage variation relative to pre-transplant weight, and served as the primary outcome for subsequent analysis.

Laboratory data included fasting plasma glucose and lipid profile (total cholesterol, high-density lipoprotein [HDL], low-density lipoprotein [LDL], triglycerides). Fasting glucose was determined by the enzymatic reference method with hexokinase, whereas total cholesterol, HDL, and triglycerides were measured using colorimetric enzymatic assays (Merk Diagnostics, Darmstadt, Germany). LDL was calculated using the Friedewald formula: LDL = total cholesterol – (HDL + triglycerides/5), provided triglyceride levels were <400 mg/dL. All measurements were performed at the institutional clinical laboratory, which adheres to standardized internal and external quality control protocols.

### Ethical considerations

The study was approved by the Research Ethics Committee of the Hospital de Clínicas de Porto Alegre. Given its retrospective design and use of secondary data from electronic medical records, the requirement for informed consent was waived. Data were accessed for research purposes after approval by the Research Ethics Committee, beginning on 04/28/2016.

### Sample size calculation

Sample size for prediction modeling was estimated with Riley et al. (2019) method (*pmsampsize function; R version 3.6.3)* [19]*.* Considering four prespecified predictors, a shrinkage factor ≥0.90, and an expected model R^2^ between 0.05 and 0.10 (based on pilot results), the recommended required sample size was 313 participants. For the binary classification analysis (>5% vs. ≤ 5% weight gain), a minimum of 20 events per variable was considered. With four prespecified predictors and an observed prevalence of 52% for weight gain >5%, the minimum required sample size for this analysis was 321 participants.

### Statistical analysis

For descriptive and stratified analyses, patients were categorized into three weight-change groups at 12 months post-transplant: weight loss >5%, weight change (gain or loss) of up to 5%, and weight gain >5%. The 5% threshold was adopted to maintain consistency with our previous study using the same cohort and was used exclusively for patient stratification [8]. Stratified comparisons were performed only for variables with at least 80% of available data. Missing data were reported for all variables as absolute frequency and percentages.

Categorical variables were described as absolute frequency (*n*) and percentage (%) and compared across groups using the Chi-square test. Continuous variables were assessed for normality using the Kolmogorov-Smirnov test. Parametric data were presented as mean ± standard deviation (SD) and analyzed using one-way analysis of variance, followed by Bonferroni post-hoc tests when appropriate. Non-parametric data were expressed as median and interquartile range and analyzed using the Kruskal-Wallis test, followed by Dunn’s post-hoc test when applicable. All statistical analyses were performed using SPSS version 20.0 (IBM, USA), with significance set at p < 0.05.

### Machine learning modeling

ML techniques were applied to develop predictive models for weight change at 12 months post-kidney-transplantation. The outcome was the percentage body weight variation, analyzed as a continuous variable. A binary classification (> 5% vs. ≤ 5% weight gain) was also explored for comparative purposes.

#### Feature selection and preprocessing.

Candidate predictors included pre-transplant BMI, recipient age at transplantation, dialysis duration, sex, donor type, and pre-transplant hypertension. Variables with more than 15% missing data were excluded. This threshold was predefined as a pragmatic criterion to balance the retention of clinically relevant predictors with the need to minimize the potential bias introduced by extensive imputation in this retrospective dataset. Only variables available at the time of transplantation were considered as candidate predictors. Therefore, post-transplant variables, including immunosuppressive drug exposure and acute rejection episodes, were not included in the modeling strategy. For the remaining predictors, missing values were imputed using the mean (continuous variables) or mode (categorical variables) estimated from the full training set. Imputation was performed before 10-fold cross-validation and was not repeated separately within each cross-validation fold. The same imputation parameters were subsequently applied to the independent testing set, which remained excluded from model development and hyperparameter tuning. The dataset was randomly divided into a training set (75%, n = 282) and a testing set (25%, n = 92).

#### Model development and evaluation.

Machine learning models evaluated included Elastic Net, Random Forest, and XGBoost. The Elastic Net algorithm was implemented for both regression (continuous outcome) and classification (binary outcome) models using the *caret* and *glmnet packages* in R *software (version 3.6.3).* Hyperparameter tuning was performed via grid search with 10-fold cross-validation. Based on cross-validated performance and model interpretability, Elastic Net with a continuous outcome was selected as the final model and applied to the independent test set (Fig 1).

**Fig 1:**
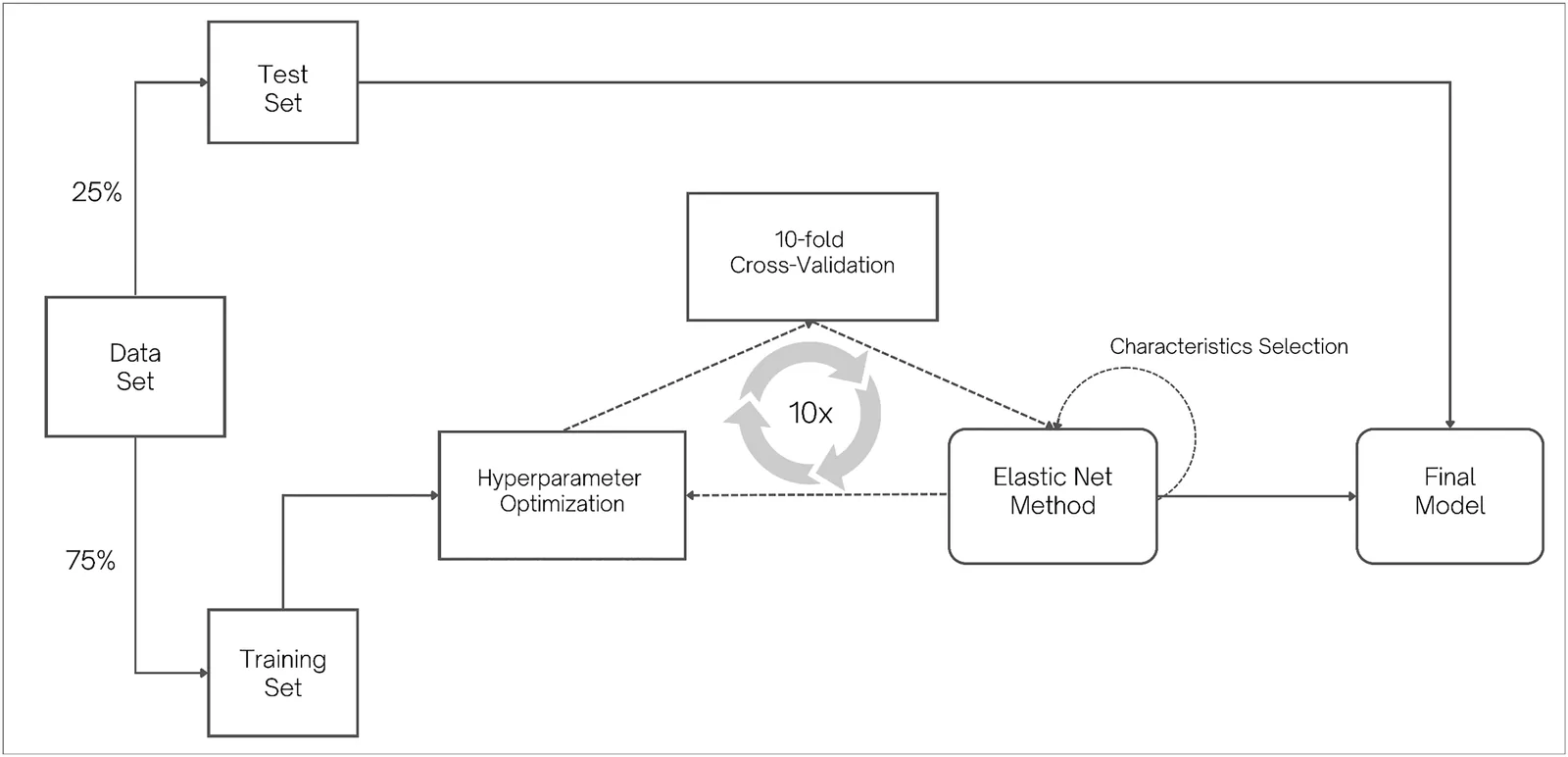
Machine Learning Protocol. Adapted from Marcon et al (2019).

Model performance was assessed using mean absolute error (MAE), root mean square error (RMSE), and coefficient of determination (R^2^) for continuous outcomes, and accuracy, area under the curve (AUC), sensitivity, and specificity for binary outcomes.

## Results

The cohort primarily comprised white patients (89.3%) and men (51.6%), with a mean age of 44 ± 13 years. Most recipients (94.9%) had undergone hemodialysis for a median of 30 months (15–60), and 71.4% received a kidney from a deceased donor. The most frequent etiologies of chronic kidney disease were undetermined (34.2%), hypertension (28.3%), and polycystic kidney disease (14.2%). Detailed demographic and clinical data are summarized in Table 1.

**Table 1:** Characteristics of patients in the pre-kidney transplant period.

Variables	Missing, *n* (%)	Value
Age (years)		44 ± 12
Male Sex, *n* (%)		193 (51.6)
Race/Ethnicity – White, *n* (%)		334 (89.3)
**Underlying Disease**		
*Undetermined*, *n* (%)		128 (34.2)
*Hypertension*, *n* (%)		106 (28.3)
*Polycystic Kidneys*, *n* (%)		53 (14.2)
*Glomerulonephritis/IgA nephropathy/Lupus*, *n* (%)		61 (16.3)
*Other Diseases*, *n* (%)		26 (7)
Previous Hypertension, *n* (%)	20 (5,3)	239 (66.9)
DM Family History, *n* (%)	157 (41,9)	103 (47.5)
Dialysis Type, *n* (%)		
*Peritoneal Dialysis*		14 (3.7)
*Hemodialysis*		355 (95)
Dialysis Time (months)		30 (15 - 60)
Donor Type – deceased n (%)		267 (71.4)
Donor sex – male, *n* (%)	5 (1.3)	187 (50.9)
Pre-transplant weight (kg)		67.1 ± 0.6
Pre-transplant BMI (kg/m^2^)		24.4 (21.9 - 27.1)
**Pre-transplant laboratory tests** *(mg/dL)*		
*Glucose*	157 (41.9)	90 (83 - 101)
*Total cholesterol*	171 (45.7)	166 (139 - 193)
*HDL*	185 (49.4)	37 (30.5 - 48)
*LDL*	201 (53.7)	94 (75 - 118)
*Triglycerides*	180 (48.1)	149 (105.7 - 214.2)

All pre-transplant laboratory parameters are expressed in mg/dL. Parametric data are presented as mean ± standard deviation (SD), and nonparametric data as median and interquartile range (P25–P75). Categorical variables are expressed as absolute number (n) and percentage (%). Unless otherwise specified in the missing column, analyses included the total sample (n = 374). **Abbreviations**: IgA: immunoglobulin A; DM: diabetes mellitus; BMI: body mass index; HDL: high-density lipoprotein; LDL: low-density lipoprotein.

Overall, 72% of recipients exhibited post-transplant weight gain, and 32% showed an increase of >10% compared with their pre-transplant body weight (Fig 2). Regarding BMI variation, the cohort showed a mean increase from 24.7 ± 4 kg/m^2^ to 26.3 ± 6 kg/m^2^ at 12 months post-transplant (Fig 3).

**Fig 2:**
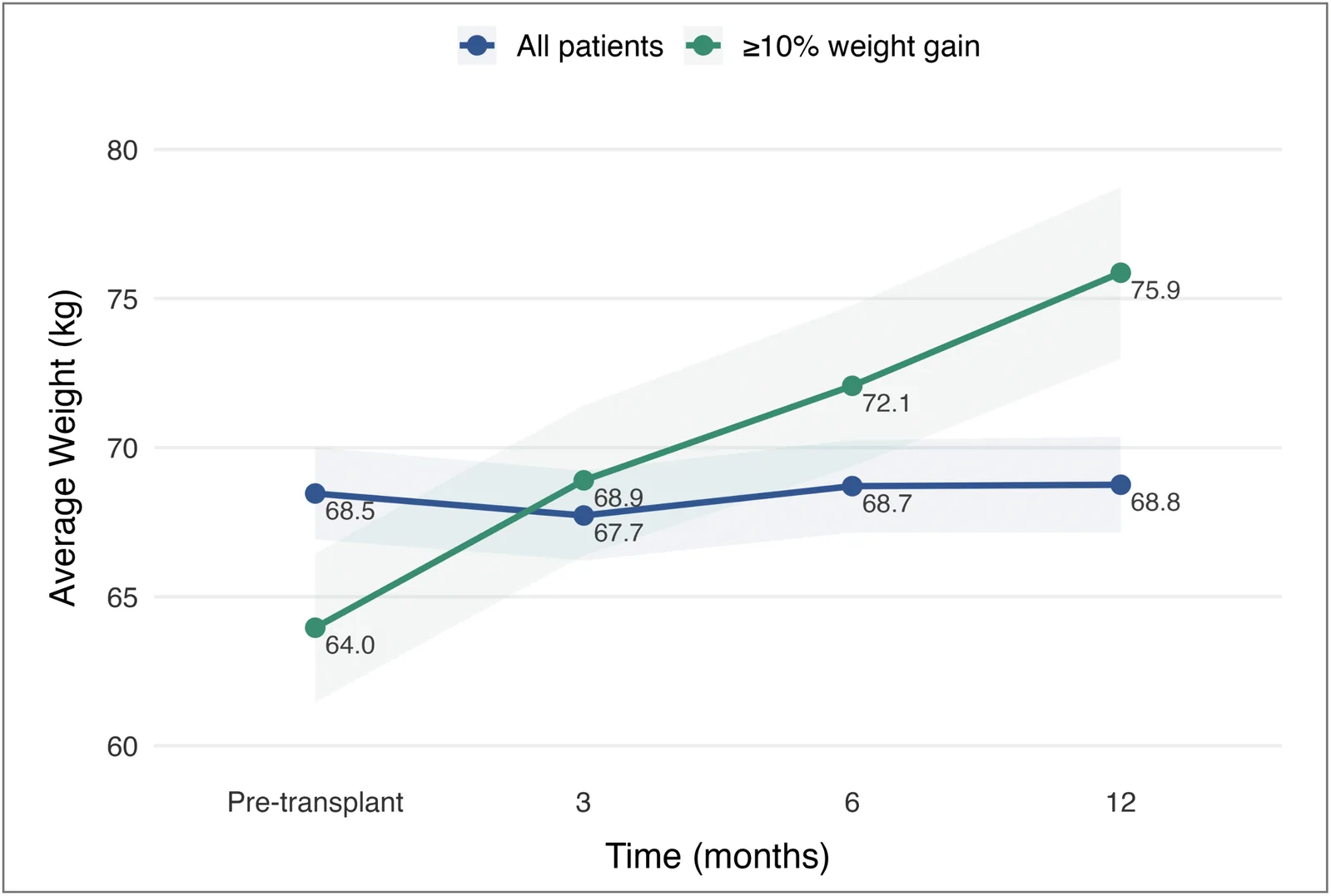
Evolution of mean body weight during the first year after kidney transplantation. Values are expressed as means (kg), and shaded areas represent 95% confidence intervals. The blue line represents the overall sample, while the green line represents patients with ≥10% weight gain relative to pre-transplant values.

**Fig 3:**
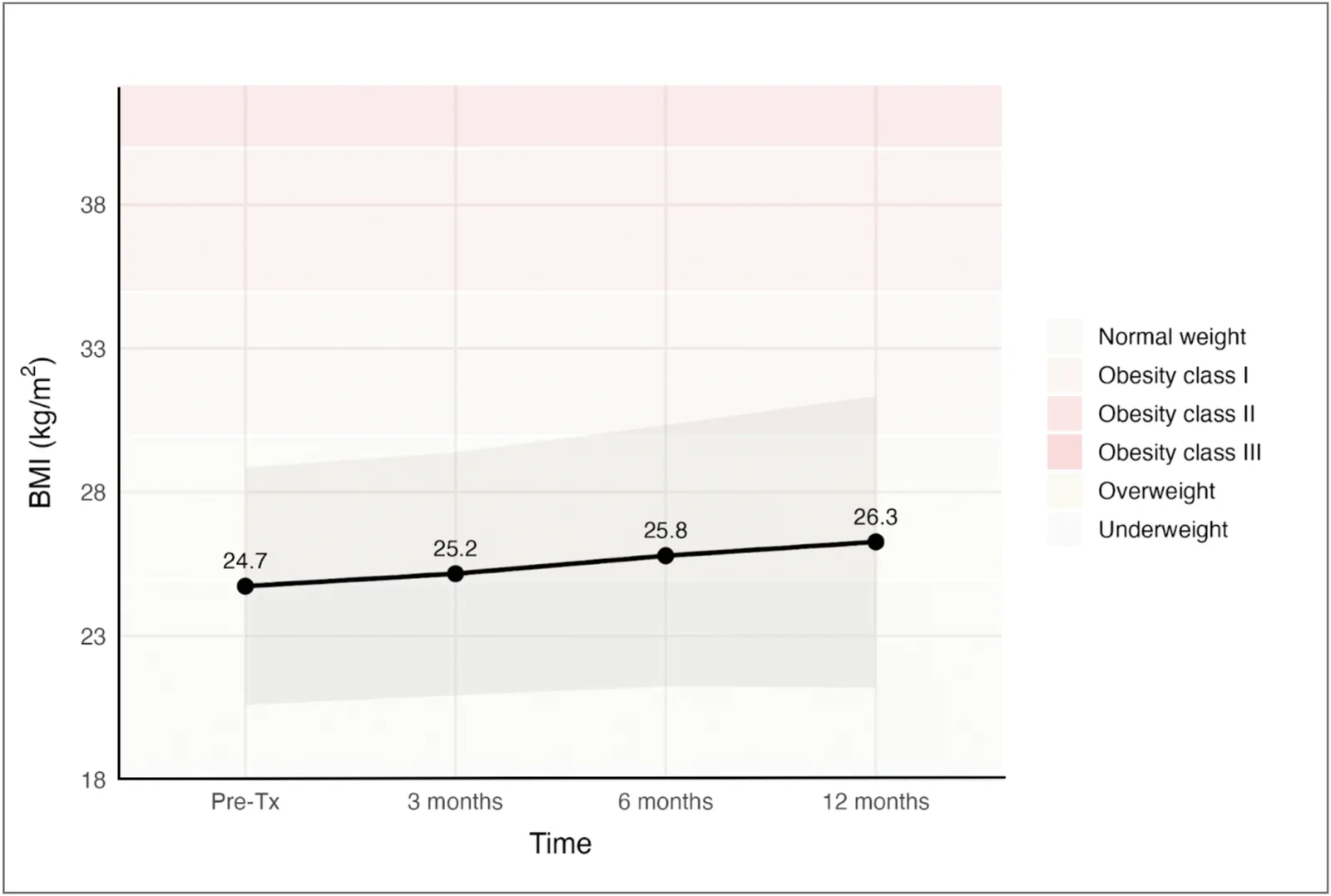
Evolution of Body Mass Index (BMI) during the first year after kidney transplantation. Values are expressed as means (kg/m^2^), and the shaded area represents the standard deviation (SD).

When stratified according to weight variation during the first year after transplantation, 184 participants presented a weight gain >5%, 124 had a weight variation ≤5%, and 46 showed a weight loss >5%. The group with weight gain >5% showed statistically significant differences compared with the other groups, with a higher proportion of female participants, younger age, lower pre-transplant weight and BMI, and a higher percentage of living donors (Supplementary Table).

### Predictive model performance

An Elastic Net regression model, selected based on cross-validated performance among the evaluated ML algorithms, was applied for variable selection using the six prespecified candidate predictors described in the Methods. Four predictors retained non-zero coefficients and composed the final model (S1 Fig and Fig 4). Following model training and testing, female sex showed the largest positive coefficient (β = 4.29), whereas transplantation from deceased donors showed a negative coefficient (β = –2.93). Higher pre-transplant BMI and older recipient age were also retained, with comparatively small negative coefficients.

**Fig 4:**
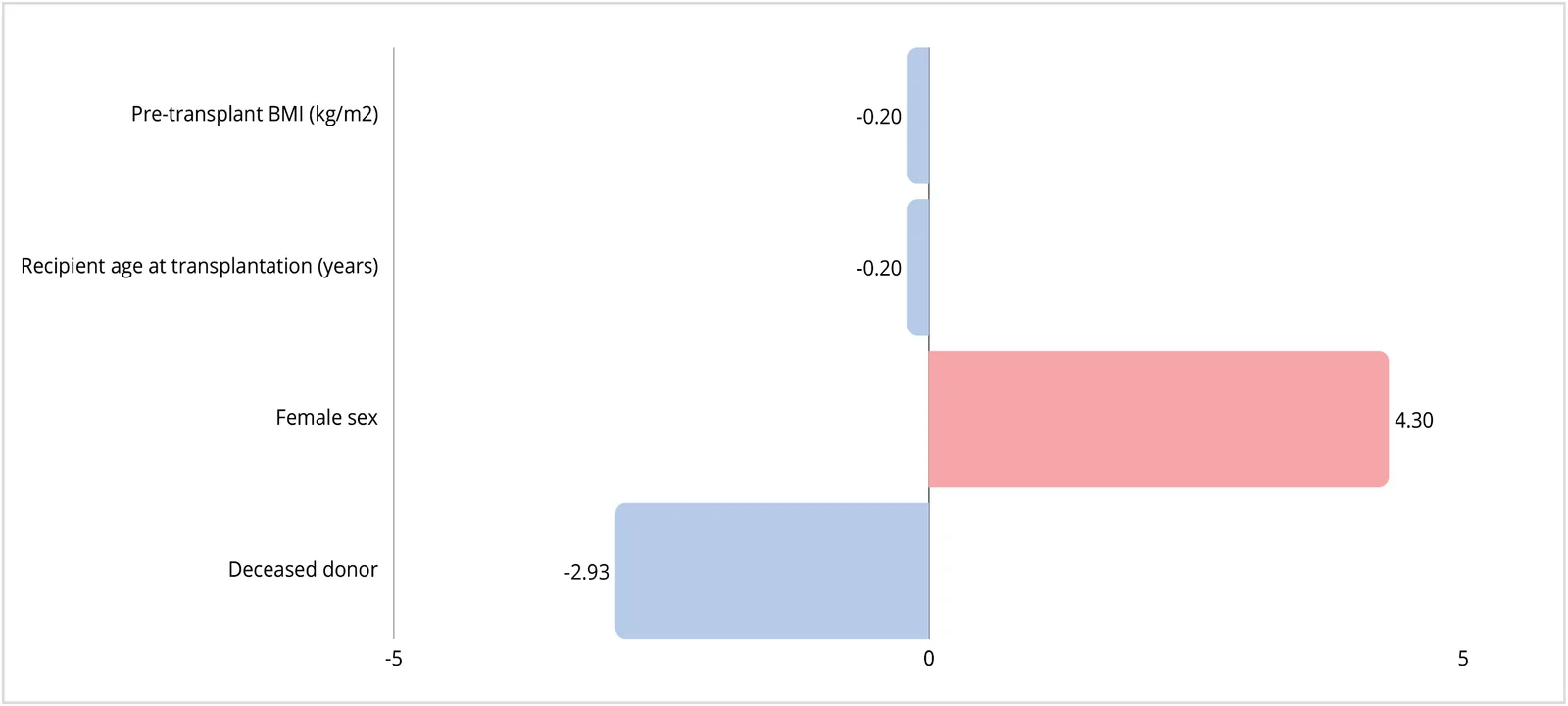
Coefficients of the Elastic Net regression model for weight change (%) at 12 months after kidney transplantation. Bars represent the estimated coefficients of each predictor retained in the model. Positive values indicate variables associated with greater predicted post-transplant weight gain, whereas negative values indicate variables associated with lower predicted post-transplant weight gain.

Model performance is summarized in Fig 5. The model explained 10% of the variance in observed weight gain (R^2^ = 0.10), indicating low but statistically significant predictive accuracy (Pearson’s r = 0.32; p = 0.01; MAE = 8.7%).

**Fig 5:**
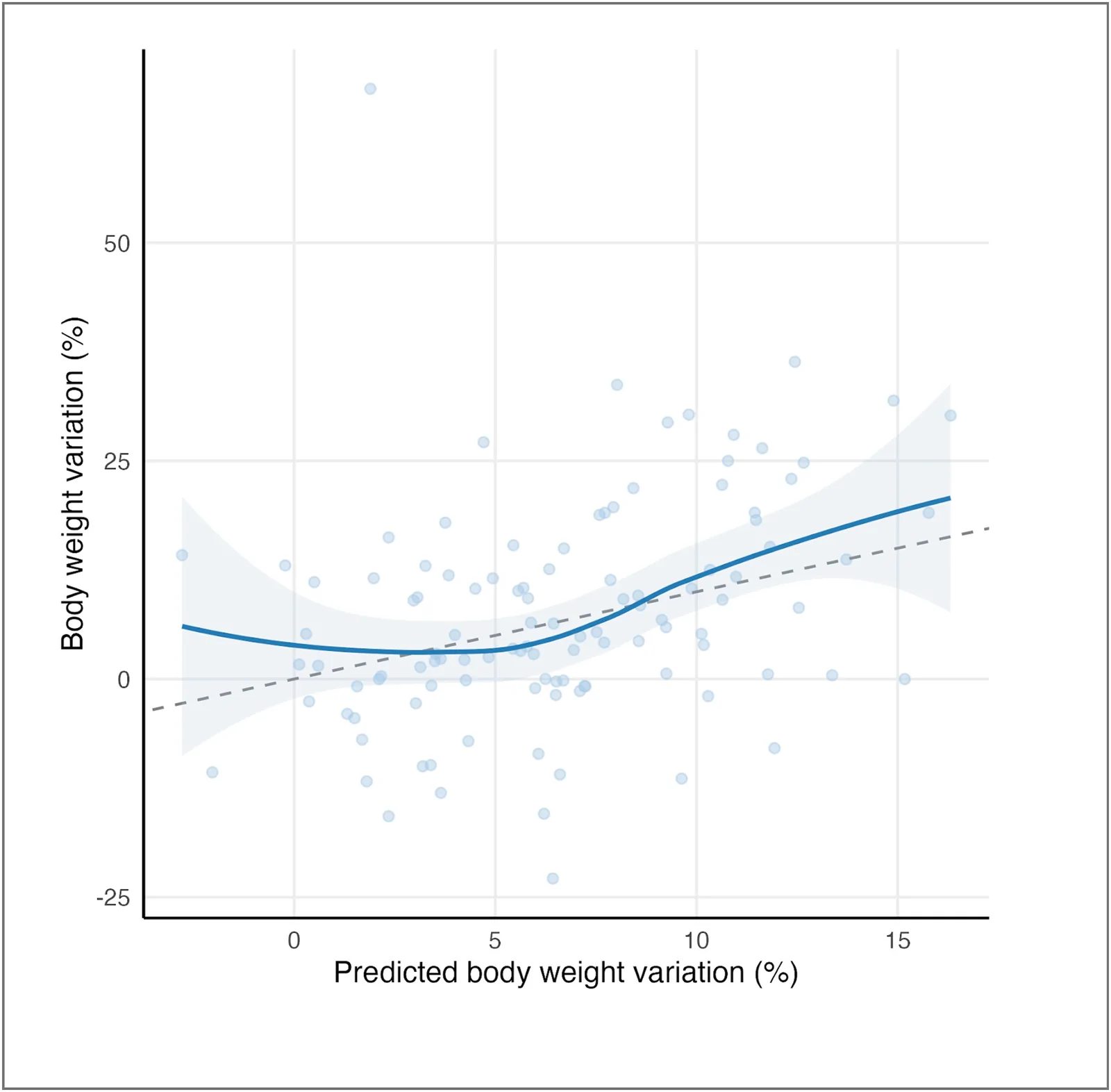
Predicted versus observed body weight variation (%) in the test set at 12 months after kidney transplantation. Scatter plot shows the relationship between the predicted and observed variation in body weight at 12 months. The dashed line represents a perfect agreement (identity line), while the solid line represents a Locally Estimated Scatterplot Smoothing (LOESS) with its 95% confidence interval (shaded area). The model showed limited predictive performance (R^2^ = 0.10).

Complementarily, the binary classification model (for weight gain >5% vs. ≤ 5%) achieved an accuracy of 68.5%, with sensitivity of 72% and a specificity of 66%. The AUC was 0.64 (S2 Fig). These values suggest limited discriminative performance, with the model showing a tendency toward higher sensitivity (detecting weight gain >5%).

## Discussion

The aim of this study was to develop a ML model to predict post-transplant weight gain, which averaged 4 ± 7.4 kg (6.6 ± 11.7%) in this cohort. The final Elastic Net model retained four predictors. Female sex showed the largest positive coefficient among the predictors retained by the model, whereas transplantation from deceased donors showed a negative coefficient. Higher pre-transplant BMI and older recipient age were also retained, with comparatively small negative coefficients.

The Elastic Net model showed limited predictive performance, explaining 10% of the variance in post-transplant weight change (R^2^ = 0.10) with a mean absolute error of 8.7%. A statistically significant, albeit modest, correlation between predicted and observed values (Pearson’s r = 0.32; p = 0.01) was observed, indicating that the model captured some variability in the outcome but had limited predictive utility. These findings support the feasibility of applying ML in this setting and provide a methodological benchmark for future studies incorporating more comprehensive clinical and pharmacological data.

According to Riley et al. (2025) and Thomassen et al. (2024), prediction models benefit from large sample sizes and comprehensive feature sets to achieve optimal stability and generalizability [20,21]. Our study met the prospectively calculated sample size requirements suggesting that model performance reflects the informational content of available variables rather than sample adequacy [19–21]. This finding emphasizes the importance of incorporating detailed clinical and pharmacological covariates in future predictive frameworks.

Predictors retained in the model, particularly donor type, should be interpreted within the methodological constraints and historical context of data collection. The finding that transplantation from deceased donors was associated with smaller weight variation is particularly relevant from a temporal standpoint. This cohort, comprising 30% of living-donor transplants, represents a transitional period in Brazil’s transplantation landscape between 2006 and 2013.

According to data from the Brazilian Transplant Registry, the proportion of deceased-donor kidney transplants increased from 46.4% in 2006 to 74.7% in 2013 and 85.3% in 2023 [22,23]. Data from the International Registry in Organ Donation and Transplantation (IRODaT) indicate that the proportion of living donors relative to deceased donors in the United States declined from 38% in 2006 to 31% in 2013 and 22% in 2023 [24]. Other high-volume transplant programs display heterogeneity mainly due to the absolute rise in total transplant activity rather than continued dependence on living donors. In Spain, for instance, the proportion of living-donor kidney transplants increased from 4% in 2006 to 12% in 2023, while the overall transplant rate rose from 48.3 to 77.7 procedures per million population [25]. We therefore interpret the association between deceased-donor transplantation and reduced weight gain as a descriptor of cohort composition rather than a generalizable finding, and refrain from causal inference given the model's limited predictive capacity. These data indicate that the model reflects patterns from a previous clinical era, reinforcing the need for predictive frameworks developed from contemporary cohorts that better represent current clinical practices and donor profiles.

A comparison with a previous study from our group that applied traditional statistical methods to the same dataset revealed both convergences and divergences [8]. Female sex consistently emerged as a significant risk factor for post-transplant weight gain. However, unlike classical analyses that associated lower pre-transplant weight with greater post-transplant weight increase, the ML model did not reproduce this pattern. Instead, it suggested mild protective effects from higher pre-transplant BMI and older recipient age. These discrepancies likely reflect the Elastic Net model’s ability to handle multicollinearity and perform variable selection in the presence of correlated predictors, as well as limitations related to the available data and potential temporal biases within the dataset. As noted by Kantidakis et al. (2023), ML approaches can perform comparably to classical statistical methods but often present lower calibration accuracy [26]. Therefore, ML techniques should be applied in a complementary and exploratory manner, particularly when clinical data are limited in detail.

Overall, the findings indicate that even with the application of advanced ML algorithms, predicting post-transplant weight gain remains challenging in the absence of detailed clinical and pharmacological information. Immunosuppressive therapy is a central determinant of metabolic changes after transplantation, influencing body weight, glucose, and lipid metabolism. In a systematic review, Kajdas et al. (2025) synthesized evidence on the metabolic outcomes of immunosuppressive therapy in kidney transplant recipients [27]. The review identified that younger recipient age, female sex, and higher creatinine levels were consistently associated with more pronounced post-transplant weight gain. Variations in calcineurin inhibitor and mammalian target of rapamycin inhibitor use, as well as exposure to corticosteroids, also contributed to interindividual differences in weight trajectories. These findings underscore the importance of incorporating pharmacological and longitudinal clinical variables when developing predictive models for metabolic outcomes in kidney transplant recipients.

The interpretation of these results requires consideration of the study's methodological constraints. The use of a retrospectively collected historical cohort (2006–2013) limits external validity, as transplant practices differ from contemporary standards. Additionally, the exclusion of post-transplant variables, including immunosuppressive drug exposure and acute rejection episodes, may have contributed to the model's limited predictive performance. Contemporary studies with more comprehensive pharmacological and clinical data may improve predictive performance. Recipients who experienced graft loss or death within the first year were excluded because assessment of the primary outcome required body weight measurements at 12 months after transplantation. Although methodologically necessary, this exclusion may have introduced survivorship bias and should be considered when interpreting the generalizability of the findings.

Despite these limitations, the study presents important strengths. To our knowledge, this is the first investigation to apply ML techniques to explore predictors of post-transplant weight gain in kidney transplant recipients. The study achieved the prospectively calculated sample size for multivariable prediction modeling, providing adequate statistical power [19]. The observed performance suggests that model limitations stem from data granularity and informational content of the available variables, rather than sample size alone. However, this cannot be concluded definitively, as model choice, residual confounding, and variability in the outcome may also have contributed.

Although the dataset holds historical relevance, it may lack sufficient predictive signal for robust modeling. Its exploratory value lies in revealing the informational limits of available data. The analysis suggests demographic and anthropometric variables alone do not fully account for post-transplant weight variability, highlighting the multifactorial nature of this outcome. The study emphasizes the need to integrate detailed clinical and pharmacological covariates, particularly immunosuppressive therapy, to enhance predictive performance. The present model should be viewed as an analytical benchmark informing future comprehensive predictive frameworks in kidney transplant research.

## Conclusion

This study evaluated the feasibility of identifying potential predictors of post-transplant weight gain in kidney transplant recipients using ML algorithms. As the first investigation to apply ML techniques specifically to this outcome in this population, it represents an important methodological contribution to transplant research. Our findings revealed that, although the Elastic Net model demonstrated limited predictive performance, it explained only a small proportion of the variance in weight outcomes. These results suggest that the available data lack sufficient informational depth to sustain a robust predictive framework. Future models should integrate detailed clinical and pharmacological variables, particularly immunosuppressive regimens and metabolic parameters, to enhance predictive capacity. This work provides a methodological basis for refining ML applications in transplant research and underscores the need for contemporary, data-rich cohorts to enable clinically meaningful prediction of post-transplant weight trajectories.

## Supporting information

S1 Table Potential predictors stratified according to body weight change one year after kidney transplantation. (PDF)

S1 File Binary Classification model. (PDF)

S1 Dataset Dataset used for the analyses presented in this study. (XLSX)

S1 Fig Predictor coefficients with their respective importance for the outcome of percentage weight gain 12 months after kidney transplantation. (TIFF)

S2 Fig ROC curve of the machine learning model for predicting weight gain >5% at 12 months after kidney transplantation. Curve plots sensitivity (true positive rate) against 1 – specificity (false positive rate). The shaded area indicates the 95% confidence interval of sensitivity, the solid line represents the mean ROC, and the dot marks the optimal cut-off determined by Youden’s index. The diagonal dashed line indicates random performance.
(TIFF)
