# Correction: Feasibility of a machine learning model for predicting weight gain after kidney transplantation machine learning and weight gain after kidney transplantation

**DOI:** 10.1371/journal.pone.0362977

**Published:** 2026-11-13

**Authors:** Júlia de Melo Cardoso de Freitas, Pedro Lemos Ballester, Elis Forcellini Pedrollo, Camila Corrêa, Rafaella Câmara Rocha Menezes, Cristiane Bauermann Leitão, Gabriela Corrêa Souza

The phrase “machine learning and weight gain after kidney transplantation” should not have been indicated in the article title. The correct title is: Feasibility of a machine learning model for predicting weight gain after kidney transplantation. The correct citation is: de Freitas JdMC, Ballester PL, Pedrollo EF, Corrêa C, Menezes RCR, Leitão CB, et al. (2026) Feasibility of a machine learning model for predicting weight gain after kidney transplantation. PLoS One 21(10): e0360501. https://doi.org/10.1371/journal.pone.0360501.
