# Supplementary material for: Feasibility of a machine learning model for predicting weight gain after kidney transplantation machine learning and weight gain after kidney transplantation
Source: PLoS One. 2026 Oct 22;21(10):e0360501. doi: 10.1371/journal.pone.0360501 (PMC13690245; doi:10.1371/journal.pone.0360501)
Supplement: Supplementary file 1 [file pone.0360501.s001.pdf]

Supplementary Table. Potential predictors stratified according to body weight change one year after kidney transplantation

**Potential predictors**	**Weight loss > 5% (*n*= 46)**	**Weight change between <5% and >5% (*n*=124)**	**Weight gain > 5% (*n*=184)**	***p value***
Age (years)	49 (41.3 - 58.5)[a]	48 (35 - 56)[a]	42 (32.8 - 52)[b]	<0.01
Male Sex, *n* (%)	22 (47.8)	78 (62.9)	78 (42.3)	<0.01
Pre-transplant weight (kg)	69 ± 11.1[a]	70.4 ± 13[a]	64.3 ± 12.5[b]	<0.001
Pre-transplant BMI (kg/m^2)	25.7 (23.7 - 28.1)[a]	24.9 (22.4 - 27.4)[a]	23.5 (21.4 - 26.3)[b]	<0.01
Donor Type - deceased, *n* (%)	38 (82.6)	100 (80.6)	116 (63)	<0.001

Data were analyzed using ANOVA followed by post hoc Bonferroni tests. Values are expressed as mean ± SD. For non-parametric data, results are presented as median and interquartile range and analyzed using the Kruskal–Wallis test followed by Dunn's post hoc test. Different letters within the same row indicate significant differences among groups ($p < 0.01$). Categorical variables are expressed as absolute numbers (n) and percentages (%) and were analyzed using the chi-square test with continuity correction. The significance level was set at $p < 0.05$. **Abbreviations**: BMI, body mass index;
