# Supplementary material for: Feasibility of a machine learning model for predicting weight gain after kidney transplantation machine learning and weight gain after kidney transplantation
Source: PLoS One. 2026 Oct 22;21(10):e0360501. doi: 10.1371/journal.pone.0360501 (PMC13690245; doi:10.1371/journal.pone.0360501)
Supplement: Supplementary file 2 [file pone.0360501.s002.pdf]

Supplementary File

Binary Classification model

A binary classification model was developed to identify recipients with clinically significant post-transplant weight gain (> 5% vs. ≤ 5% at 12 months). The Elastic Net classifier achieved an accuracy of 68.5%, with sensitivity of 72% and specificity of 66%, yielding an area under the receiver operating characteristic curve (AUC) of 0.64 and an optimal Youden threshold of approximately 0.54 (Fig S2). These metrics indicate limited discriminative performance, slightly above random classification, with a mild tendency toward higher sensitivity, favoring the identification of individuals with greater post-transplant weight gain.
